# Remote learning and physical activity in medical students from Latin America: a cross-sectional study during the first wave of the COVID-19 pandemic

**DOI:** 10.3389/fspor.2025.1684127

**Published:** 2026-01-12

**Authors:** Mario J. Valladares-Garrido, Renzo Acosta-Porzoliz, Alejandro Juarez-Ubillus, Milagros Diaz-Torres, Ludwing A. Zeta Solis, David Astudillo Rueda, Fatima Jiménez-Mozo, C. Ichiro Peralta Chiguala, Christopher G. Valdiviezo-Morales, E. Sebastian Benavides Alburqueque, Estrella Christabel Porras Núñez, Víctor J. Vera-Ponce, César J. Pereira-Victorio, Carlos Culquichicón

**Affiliations:** 1 Escuela de Medicina Humana, Universidad Señor de Sipán, Chiclayo, Peru; 2 Facultad de Medicina Humana, Universidad Peruana Cayetano Heredia, Lima, Peru; 3 EpiHealth Research Center for Epidemiology and Public Health, Lima, Peru; 4 Facultad de Medicina Humana, Universidad de San Martín de Porres, Chiclayo, Peru; 5 Facultad de Medicina, Universidad Nacional de Piura, Piura, Peru; 6 Escuela de Medicina, Universidad Cesar Vallejo, Piura, Peru; 7 Universidad Nacional Federico Villarreal, Lima, Perú; 8 Facultad de Medicina (FAMED), Universidad Nacional Toribio Rodríguez de Mendoza de Amazonas (UNTRM), Amazonas, Peru; 9 Facultad de Medicina, Universidad Continental, Lima, Perú; 10 University of Washington, School of Public Health, Seattle, WA, United States

**Keywords:** COVID-19, cross-sectional study, Latin America, medical students, physical activity, remote learning

## Abstract

**Objective:**

To evaluate the association between remote learning and physical activity levels among medical students in Latin America during the first wave of the COVID-19 pandemic.

**Methods:**

Multicenter cross-sectional study conducted via an online survey during 2020, targeting medical students from multiple Latin American countries. Sociodemographic, academic, mental health, and physical activity data were collected using the International Physical Activity Questionnaire (IPAQ-S). Remote learning was the main exposure variable, and low physical activity level was the outcome. Poisson regression models with robust variance were used to estimate adjusted prevalence ratios (PR) and 95% confidence intervals (95% CI).

**Results:**

Among 2018 medical students, the prevalence of low physical activity was 54.5% (95% CI: 52.3–56.7). In the multivariable analysis, remote/virtual learning was associated with a lower prevalence of high physical activity (PR = 0.81). Other factors associated with lower prevalence included female sex (PR = 0.89), obesity (PR = 0.69), trust in the government (PR = 0.79), and high-risk tobacco use (PR = 0.80). Factors associated with higher prevalence included perceiving the pandemic as mild/not serious (PR = 1.40), prior COVID-19 diagnosis (PR = 1.21), studying at a private university (PR = 1.17), and having diabetes (PR = 1.59).

**Conclusions:**

Remote learning during the pandemic was associated with lower physical activity levels among Latin American medical students. These findings highlight the need for university-based interventions to promote physical activity, particularly in prolonged distance learning contexts.

## 1 Introduction

The COVID-19 pandemic led the global population to adopt preventive measures such as hygiene practices, social distancing, and home isolation, in order to control the spread of the virus and flatten the curve (1). Due to this new social context, daily life activities were drastically affected, including work routines, recreational activities, and educational classes in schools and universities (2). These public health measures implemented to prevent the spread of COVID-19 posed a significant global challenge in maintaining an active physical state among individuals due to the lack of physical activity and exercise, especially among students (3). This, in turn, induced muscle loss, neuromuscular damage, insulin resistance, and fat deposition, thereby impairing their metabolism (3).

Previous studies conducted during the first wave of the pandemic estimated that 48.2%, 63%, and 65% of medical students in Pakistan, Bahrain, and Italy, respectively, experienced a decrease in their level of physical activity (4–6). In Latin America, 48.5%, 67%, and 66.2% of students from Colombia, Brazil, and Peru, respectively, reported the same situation (2, 7, 8). This behavior can negatively impact health by increasing body fat, which in some cases leads to a loss of self-esteem and difficulty in maintaining one's lifestyle (5, 9). Furthermore, sedentary behavior over prolonged periods can lead to physiological adaptations that impair and reduce the physical level of mitochondrial function, thereby exacerbating any existing health conditions (5).

Additionally, a study conducted in Peru by Miranda-Larios at the Universidad Ricardo Palma examined participation in physical activity and attendance in virtual classes among medical students. The author reported that 32% of students presented low levels of physical activity and dedicated approximately 20% of their daily time to online classes (10). In Argentina and Colombia, a decrease in physical activity of 39% and 38.8%, respectively, was reported, which was attributed to the time students devoted to their studies and virtual classes (7, 11). While remote education has allowed for the continuation of medical learning, it may have negative effects on physical activity, as both physical and mental health could be influenced and deteriorated as a result of these unusual changes (12).

However, although previous investigations have been conducted on remote education and physical activity in medical students, the evidence regarding this research question remains inconclusive.

First, previous evidence has not specifically measured the association of interest (type of remote education and physical activity) in medical students, much less in university students during the context of the COVID-19 pandemic (7, 11). Second, most studies associate physical activity with students' mental health (13, 14). Third, most studies have not considered the population of students from various medical schools in Latin America (15, 16). Fourth, previous studies have not evaluated potential confounding variables (being single, religion, being a parent, participation in extracurricular groups, having a disease, alcohol, tobacco, and drug use, etc.), which may result in potential information bias (7, 17). Fifth, similar investigations have been designed with a descriptive approach and lack sufficient biostatistical rigor (18, 19). Sixth, there is insufficient research focused primarily on our main variable in medical students (7, 20).

Several preventive programs and interventions have been implemented in Latin America to promote healthier lifestyles among university students, including campus-based physical activity initiatives, digital exercise platforms, and community campaigns encouraging movement and recreational sports (21–23). These strategies have shown varying degrees of impact, highlighting the importance of tailoring interventions to local resources and cultural contexts (24). Moreover, studies comparing university students across Latin American countries have reported substantial differences in physical activity patterns, shaped by socioeconomic conditions, academic environments, cultural norms, and access to safe spaces for exercise (25, 26). Evidence from countries such as Brazil, Colombia, Mexico, and Chile demonstrates heterogeneous levels of physical activity and diverse barriers to maintaining active lifestyles during the COVID-19 pandemic (27–30). Incorporating this regional variability underscores the need for a multicountry approach to better understand the determinants of physical activity in this population.

Based on the above, the objective of this research was to identify the association between remote learning and the level of physical activity in medical students from 13 Latin American countries during the first wave of COVID-19, as well as other factors potentially associated with physical activity in this student population.

## 2 Materials and methods

### 2.1 Study design

Analytical cross-sectional study based on secondary data analysis, obtained from medical students in 13 Latin American countries during the first wave of the COVID-19 pandemic. The primary study aimed to assess the association between resilience and post-traumatic stress disorder due to COVID-19, while this secondary analysis focused on determining the association between remote learning and the level of physical activity in this population group.

### 2.2 Population, sample, and sampling

The study population included medical students enrolled in universities from Argentina, Bolivia, Brazil, Chile, Colombia, Cuba, Ecuador, El Salvador, Mexico, Paraguay, Peru, and Venezuela. Universities were selected based on the presence of scientific societies of medical students or other academic groups with active participation during the pandemic.

In the primary study, students enrolled in the 2020-I academic term, aged over 18 years, and who provided informed consent were included. Those who did not complete the key variables in the questionnaire or who did not agree to participate in the research were excluded. For this secondary analysis, only records with complete information on resilience and associated factors were considered.

The sample size calculation in the primary study was performed considering a statistical power of 80%, a significance level of 95%, and an expected correlation between resilience and post-traumatic stress of 0.108, based on previous studies in medical students. A minimum sample size of 670 students was estimated, adjusted for a 40% rejection rate and a 35% loss, resulting in a final sample size of 2,680 participants.

Sampling was conducted using a non-probabilistic snowball method, in which participants were invited to complete an online questionnaire and to share it with their fellow students. Data collection took place between June 15 and September 15, 2020, during a period of mandatory confinement in most of the participating countries.

### 2.3 Statistical power

A statistical power of 84.1% was estimated to assess the association between virtual academic workload and physical activity level in medical students from Latin America. For this calculation, the proportions of high physical activity in two groups were considered: those with in-person academic workload (*p*_1_ = 0.61) and those with virtual academic workload (*p*_2_ = 0.53). Additionally, the corresponding sample sizes were taken into account, with *n*_1_ = 425 students in the in-person group and *n*_2_ = 1,593 in the virtual group.

### 2.4 Instruments

#### 2.4.1 IPAQ physical activity questionnaire (IPAQ-S, short form): 9-item short version

An available instrument for assessing physical activity over a 7-day period consists of 9 questions in its abbreviated version, covering four domains of physical activity (leisure time, household activities, work, and transportation). Each of these domains is subdivided into four types of physical activity (sitting, walking, moderate activities, and vigorous activities) (31). A common method has been used to validate this questionnaire by correlating self-reported activity data with data obtained from objective measurement devices during the same period. Additionally, absolute differences between objective and self-reported measurements can be calculated as a secondary method, as validity results may vary depending on the index and the standard of objective measurement used (32). This instrument has been applied in Latin American populations, specifically in Argentina, Brazil, Chile, Colombia, Costa Rica, Ecuador, Peru, and Venezuela, where paired Student's t-tests showed fluctuations with values ranging from 33.4 to 35.4 (33). Previously, this instrument was used to assess physical activity in medical students during distance education (34). Likewise, it was employed in the context of COVID-19 among medical students (16, 35).

#### 2.4.2 Substance Use questionnaire

A Spanish-validated questionnaire used to identify harmful levels of substance use. It consists of 8 items regarding recent and lifetime use of 10 substances (tobacco, alcohol, cannabis, cocaine, amphetamines or other stimulants, anxiolytics, hallucinogens, inhalants, and other drugs) (36). The questionnaire assesses various domains using closed-ended multiple-choice questions for each substance, and total scores are calculated for the test (36). A cutoff score between 4 and 26 has been established, with values in this range indicating moderate-risk or harmful use, respectively (36). Scores above 27 indicate a high risk of dependence, with a high likelihood of experiencing relationship, health, social, economic, or legal problems as a result of substance use (36). This instrument has been used to assess Latin American university students, particularly in the Mexican population, showing Cronbach's alpha values ranging from 0.85 to 0.87 (37). It has previously been used to evaluate university populations during distance education (38). Likewise, it was employed in the context of COVID-19 with medical students (39, 40).

### 2.5 Variables

The dependent variable was the level of physical activity, operationally defined as the self-reported frequency and intensity of physical activity performed by medical students, according to the International Physical Activity Questionnaire, short 9-item version. It was categorized into three levels: low, moderate, and high, in accordance with the criteria established in the instrument.

The main independent variable was remote learning, operationally defined as the self-reported experience of receiving all or the vast majority of academic coursework through virtual platforms during the semester in which the survey was conducted, with minimal or no in-person attendance. This was assessed with the question: “Are you currently taking your academic courses remotely/virtually?” (Response options: Yes/No). Given the multicenter nature of the study, the extent and format of remote learning could vary across universities and countries due to local public health regulations and institutional policies; therefore, classification relied on each participant's perception of their predominant learning modality.

The independent variables of the study included age in years and sex (male and female), the type of university, distinguishing between public and private institutions, as well as the year of study, classified from the first to the seventh year of the medical program. Regarding personal and family variables, participation in extracurricular groups was assessed, along with marital status, classified as single and not single, and religion, categorized as none, Catholic, and non-Catholic. Parenthood and the number of family members were also considered. With respect to nutritional status, body mass index was included and categorized as underweight/normal, overweight, and obese.

In terms of consumption habits and health, self-reported alcohol and tobacco use were assessed, categorized as low and moderate/high. Additionally, the presence of hypertension and diabetes mellitus was included, both treated as dichotomous variables based on self-reported medical diagnosis. To evaluate preventive measures related to the pandemic, compliance with social isolation was recorded, as well as the perception of the severity of the pandemic, the latter classified as very serious, serious, and mild/not serious. Confidence in the government's ability to manage the COVID-19 pandemic was also analyzed.

On the other hand, personal history of COVID-19, the presence of a diagnosis in a family member, and the loss of a family member due to the virus were included. Finally, a personal history of mental health was considered, based on self-reported previous diagnosis of psychological disorders.

### 2.6 Procedures

The study was approved by the Ethics Committee for the primary research. Subsequently, a working team was established in collaboration with the Latin American Federation of Scientific Societies of Medical Students (FELSOCEM), ensuring broad coverage of participating university sites across Latin America. Coordinators were recruited at each study site and trained in data collection procedures, the use of the REDCap platform, and ethical principles in human research. Data collection was carried out through a structured online questionnaire designed using the REDCap (Research Electronic Data Capture) platform, ensuring data security and traceability. A snowball sampling method was employed to disseminate the study, contacting student representatives from universities in various Latin American countries, who promoted participation through social media and academic platforms. Before completing the questionnaire, participants were informed about the study objectives, the voluntary nature of their participation, and the confidentiality of their responses. They then completed the questionnaire, which had an estimated completion time of approximately 20 min. After data collection, a data cleaning process was conducted to eliminate incomplete or inconsistent records, ensuring the validity of the analyses. The data were stored on a secure server with access restricted to the principal investigators. Data anonymization measures were implemented to ensure compliance with data protection standards and research ethics.

### 2.7 Analysis plan

For data analysis, a descriptive summary of the variables was conducted. Categorical variables were expressed as absolute frequencies and percentages, while numerical variables were presented as mean and standard deviation (SD) in the case of normal distribution, or as median and interquartile range (IQR) in the case of non-normal distribution, based on analytical and descriptive tests.

In the bivariate analysis, the association between physical activity level and virtual academic workload was evaluated, along with other socio-educational characteristics of the participants. For categorical variables, the chi-square test of independence was used, or Fisher's exact test when expected frequencies were less than 5. For continuous variables, the Student's *t*-test was applied in cases of normal distribution, and the Mann–Whitney *U* test for non-normally distributed data.

For simple and multiple regression analyses, Generalized Linear Models (GLM) with a Poisson distribution family and robust variance were used, estimating prevalence ratios (PR) with 95% confidence intervals (95% CI). In the multiple model, the association of interest (virtual academic workload vs. physical activity level) was adjusted for all secondary independent variables in order to evaluate changes in the direction and strength of the association compared to the simple regression model. Collinearity among independent variables was assessed. Given the multicenter design of the study and potential intra-cluster correlation by country, clustering adjustment by country of origin was incorporated to ensure more accurate estimates.

Statistical analysis was conducted using Stata software version 18.0 (StataCorp LP, College Station, TX, USA), considering a statistical significance level of *p* < 0.05 for all hypothesis tests.

### 2.8 Ethical considerations

Ethical approval for the primary study was obtained from the COVID-19 Research Ethics Committee of the Seguro Social de Salud (EsSalud), Lima-Peru. Participant confidentiality was ensured through data anonymization and restricted access to the database. All participants provided digital informed consent, guaranteeing their voluntary participation and the right to withdraw at any time. Data collection was conducted using REDCap, in compliance with data protection regulations.

## 3 Results

### 3.1 Socioeducational and psychosocial characteristics of medical students

Among 2,018 medical students, the median age was 21 years (IQR: 19–23 years), with the majority being female (64.6%) and enrolled in public universities (52.0%). Regarding the year of study, first- and second-year students represented the highest proportions (19.3% and 19.5%, respectively). A total of 34.9% of students reported belonging to an academic group in addition to their studies. Moreover, the vast majority of participants were single (97.1%), did not have children (96.8%), and Catholicism was the most frequently reported religion (64.6%). In terms of health characteristics, 24.9% of students were overweight and 4.9% were obese. Moderate/high tobacco use was reported by 20.5% of students, while 11.0% reported moderate/high alcohol use. Hypertension and diabetes mellitus were reported by 1.8% and 1.2% of students, respectively. In the context of the COVID-19 pandemic, 52.6% of participants had a family member diagnosed with COVID-19, and 10.2% reported the loss of a family member due to the virus. A total of 62.0% considered the pandemic a very serious event, while 77.2% expressed distrust in the government's ability to manage the health crisis. Regarding academic workload, 78.9% of students reported having a remote learning (95% CI: 77.09–80.70). Finally, concerning physical activity, 36.6% had a low level of activity, and only 8.9% reported a moderate level of physical activity. The prevalence of high physical activity was 54.5% (95% CI: 52.31–56.70) Table 1.

**Table 1:** Characteristics of medical students (*n* = 2,019).

Characteristics	*N* (%)
Age (years)^a^	21 (19–23)
Sex	
Male	715 (35.4)
Female	1,303 (64.6)
Type of university	
Public	1,049 (52.0)
Private	969 (48.0)
Year of study	
First	389 (19.3)
Second	394 (19.5)
Third	364 (18.0)
Fourth	352 (17.4)
Fifth	292 (14.5)
Sixth	128 (6.3)
Seventh	99 (4.9)
Belongs to an extracurricular group	
No	1,314 (65.1)
Yes	704 (34.9)
Marital status: Single	
No	59 (2.9)
Yes	1,960 (97.1)
Religion	
None	491 (24.3)
Catholic	1 305 (64.6)
Non-Catholic	223 (11.1)
Has children	
No	1,954 (96.8)
Yes	65 (3.2)
Number of family members	4 (3–5)
BMI (categorized)	
Underweight/Normal	1,418 (70.3)
Overweight	502 (24.9)
Obesity	98 (4.9)
Alcohol consumption	
Low	827 (89.0)
Moderate/High	102 (11.0)
Tobacco use	
Low	739 (79.5)
Moderate/High	190 (20.5)
Arterial hypertension	
No	1,983 (98.2)
Yes	36 (1.8)
Diabetes mellitus	
No	1,995 (98.8)
Yes	24 (1.2)
Compliance with social isolation measures	
No	106 (5.3)
Yes	1,913 (94.8)
Perception of the severity of the pandemic	
Very serious	1,251 (62.0)
Serious	735 (36.4)
Mild/Not serious	33 (1.6)
Confidence in the government's ability to manage the COVID-19 pandemic	
No	1,558 (77.2)
Yes	461 (22.8)
Personal history of COVID-19	
No	1,948 (96.5)
Yes	71 (3.5)
Family member diagnosed with COVID-19	
No	958 (47.5)
Yes	1,061 (52.6)
Family member deceased due to COVID-19	
No	1,813 (89.8)
Yes	206 (10.2)
Personal history of mental health conditions	
No	1,792 (88.8)
Yes	227 (11.2)
Remote learning	
No	425 (21.1)
Yes	1,593 (78.9)
Physical activity	
Low	738 (36.6)
Moderate	180 (8.9)
High	1,100 (54.5)

a Median (25th–75th percentile).

### 3.2 Remote learning and other factors associated with high levels of physical activity: bivariate analysis

Students who were engaged in remote or virtual academic workloads had a lower proportion of high physical activity levels compared to those with in-person academic workloads (52.67% vs. 61.4%; *p* = 0.001) Table 2.

**Table 2:** Remote learning and other factors associated with physical activity, in bivariate analysis.

Variables	Physical activity		*p* *
	Low/moderate (*n* = 918)	High (*n* = 1,100)	
	*n* (%)	*n* (%)	
Age (years)***	21 (19–23)	21 (19–23)	0.188**
Sex			0.038
Male	303 (42.4)	412 (57.6)	
Female	615 (47.2)	688 (52.8)	
Type of university			0.291
Public	489 (46.6)	560 (53.4)	
Private	429 (44.3)	540 (55.7)	
Belongs to an extracurricular group			0.622
No	603 (45.9)	711 (54.1)	
Yes	315 (44.7)	389 (55.3)	
Marital status: Single			0.966
No	27 (45.8)	32 (54.2)	
Yes	891 (45.5)	1,068 (54.5)	
Religion			0.639
None	223 (45.4)	268 (54.6)	
Catholic	600 (46.0)	704 (54.0)	
Non-Catholic	95 (42.6)	128 (57.4)	
Has children			0.385
No	885 (45.3)	1,068 (54.7)	
Yes	33 (50.8)	32 (49.2)	
Number of family members	4.32 ± 1.68	4.29 ± 1.74	0.701
BMI (categorized)			0.008
Underweight/Normal	619 (43.7)	799 (56.4)	
Overweight	242 (48.2)	260 (51.8)	
Obesity	57 (58.2)	41 (41.8)	
Alcohol consumption			0.312
Low	394 (47.6)	433 (52.4)	
Moderate/High	54 (52.9)	48 (47.1)	
Tobacco use			0.008
Low	340 (46.0)	399 (54.0)	
Moderate/High	108 (56.8)	82 (43.2)	
Arterial hypertension			0.376
No	899 (45.4)	1,083 (54.6)	
Yes	19 (52.8)	17 (47.2)	
Diabetes mellitus			0.429
No	909 (45.6)	1,085 (54.4)	
Yes	9 (37.5)	15 (62.5)	
Compliance with social isolation measures			0.174
No	55 (51.9)	51 (48.1)	
Yes	863 (45.1)	1,049 (54.9)	
Perception of the severity of the pandemic			0.097
Very serious	568 (45.4)	682 (54.6)	
Serious	341 (46.4)	394 (53.6)	
Mild/Not serious	9 (27.3)	24 (72.7)	
Confidence in the government's ability to manage the COVID-19 pandemic			0.174
No	696 (44.7)	862 (55.3)	
Yes	222 (48.3)	238 (51.7)	
Personal history of COVID-19			0.865
No	885 (45.5)	1,062 (54.6)	
Yes	33 (46.5)	38 (53.5)	
Family member diagnosed with COVID-19			0.217
No	422 (44.1)	536 (56.0)	
Yes	496 (46.8)	564 (53.2)	
Family member deceased due to COVID-19			0.796
No	823 (45.4)	990 (54.6)	
Yes	95 (46.3)	110 (53.7)	
Personal history of mental health conditions			0.129
No	804 (44.9)	987 (55.1)	
Yes	114 (50.2)	113 (49.8)	
Remote learning			0.001
No	164 (38.6)	261 (61.4)	
Yes	754 (47.3)	839 (52.7)	

* *p*-value for categorical variables calculated using the Chi-square test.

** *p*-value for numerical variables calculated using the Mann–Whitney *U* test.

*** Median—interquartile range.

Additionally, a significant association was observed between country of origin and physical activity level (*p* < 0.001). Students from Mexico (75%), Bolivia (65.8%), and Venezuela (64.7%) had higher proportions of high physical activity levels compared to those from Argentina (45.07%) and Brazil (52.63%). Male students had a lower proportion of high physical activity levels compared to female students (52.8% vs. 57.6%; *p* = 0.039). The proportion of high physical activity levels among obese students was lower compared to students with normal weight (41.8% vs. 56.4%; *p* = 0.020). Students at moderate/high risk of tobacco use had a lower proportion of high physical activity levels compared to those not at risk (43.2% vs. 54.0%; *p* = 0.008). Table 2.

### 3.3 Remote learning and other factors associated with high levels of physical activity: simple and multiple regression analysis

In the simple regression model, we found that having a remote academic workload was associated with a 14% lower prevalence of high physical activity levels (PR: 0.86; 95% CI: 0.75–0.96). In the multiple regression model, adjusted for confounders, the direction and magnitude of the association of interest remained consistent. Students with remote or virtual academic workloads had a 19% lower prevalence of high physical activity levels compared to those without (PR: 0.81; 95% CI: 0.72–0.92) (Table 3).

**Table 3:** Remote learning and other factors associated with physical activity, according to bivariate and multivariate regression analysis.

Characteristics	Physical activity					
	Bivariate regression			Multivariate regression		
	PR	95% CI	*p* *	PR	95% CI	*p* *
Age (years)*	1.00	0.99–1.01	0.526	1.01	0.98–1.04	0.295
Sex						
Male	Ref.			Ref.		
Female	0.92	0.99–0-95	<0.001	0.89	0.82–0.96	0.002
Type of university						
Public	Ref.			Ref.		
Private	1.04	0.88–1.24	0.610	1.17	1.02–1.33	0.021
Belongs to an extracurricular group						
No	Ref.			Ref.		
Yes	1.00	0.93–1.12	0.657	1.10	0.98–1.22	0.104
Marital status: Single						
No	Ref.			Ref.		
Yes	1.01	0.83–1.22	0.955	1.00	0.74–1.36	0.981
Religion						
None	Ref.			Ref.		
Catholic	0.99	0.92–1.06	0.779	0.95	0.87–1.04	0.295
Non-Catholic	1.05	0.91–1.21	0.489	1.08	0.89–1.30	0.444
Has children						
No	Ref.			Ref.		
Yes	0.90	0.74–1.10	0.292	0.68	0.28–1.62	0.381
Number of family members	1.00	0.98–1.01	0.624	1.00	0.97–1.02	0.745
BMI (categorized)						
Underweight/Normal	Ref.			Ref.		
Overweight	0.92	0.87–0.97	0.001	0.92	0.80–1.06	0.243
Obesity	0.74	0.65–0.85	<0.001	0.69	0.51–0.93	0.015
Alcohol consumption						
Low	Ref.			Ref.		
Moderate/High	0.90	0.81–0.99	0.037	1.01	0.93–1.10	0.767
Tobacco use						
Low	Ref.			Ref.		
Moderate/High	0.80	0.62–1.02	0.077	0.80	0.63–0.99	0.048
Arterial hypertension						
No	Ref.			Ref.		
Yes	0.86	0.62–1.19	0.376	0.74	0.45–1.21	0.230
Diabetes mellitus						
No	Ref.			Ref.		
Yes	1.15	0.85–1.55	0.362	1.59	1.16–2.18	0.004
Compliance with social isolation measures						
No	Ref.			Ref.		
Yes	1.14	0.94–1.39	0.184	1.08	0.77–1.52	0.656
Perception of the severity of the pandemic						
Very serious	Ref.			Ref.		
Serious	0.98	0.92–1.05	0.600	0.96	0.92–1.01	0.123
Mild/Not serious	1.33	1.15–1.54	<0.001	1.40	1.09–1.79	0.008
Confidence in the government's ability to manage the COVID-19 pandemic						
No	Ref.			Ref.		
Yes	0.94	0.86–1.03	0.156	0.79	0.70–0.89	<0.001
Personal history of COVID-19						
No	Ref.			Ref.		
Yes	0.98	0.74–1.30	0.893	1.21	1.03–1.41	0.021
Family member diagnosed with COVID-19						
No	Ref.			Ref.		
Yes	0.95	0.89–1.02	0.163	1.02	0.93–1.12	0.727
Family member deceased due to COVID-19						
No	Ref.			Ref.		
Yes	0.99	0.80–1.21	0.899	0.92	0.74–1.14	0.456
Personal history of mental health conditions						
No	Ref.			Ref.		
Yes	0.90	0.80–1.02	0.088	0.95	0.83–1.08	0.425
Remote learning						
No	Ref.			Ref.		
Yes	0.86	0.75–0.98	0.019	0.81	0.72–0.92	0.001

* *p*-values obtained using Generalized Linear Models (GLM), Poisson family, log link function, robust variance.

Additionally, we identified other factors associated with high levels of physical activity in the final multiple regression model. Female students had an 11% lower prevalence of high physical activity levels compared to male students (PR: 0.89; 95% CI: 0.82–0.96). Perceiving the pandemic as mild/non-serious was associated with a 40% higher prevalence of high physical activity levels (PR: 1.40; 95% CI: 1.09–1.79). Students with diabetes had a 59% higher prevalence of high physical activity levels (PR: 1.59; 95% CI: 1.16–2.18). Students diagnosed with COVID-19 had a 21% higher prevalence of high physical activity levels compared to those without a diagnosis (PR: 1.21; 95% CI: 1.03–1.41). Studying at a private university instead of a public one was associated with a 17% higher prevalence of high physical activity levels (PR: 1.17; 95% CI: 1.02–1.32). In contrast, obese students had a 31% lower prevalence of high physical activity levels (PR: 0.69; 95% CI: 0.51–0.93). Having confidence in the government's ability to manage the COVID-19 pandemic was associated with a 21% lower prevalence of high physical activity levels (PR: 0.79; 95% CI: 0.70–0.89). Students at high risk for tobacco use had a 20% lower prevalence of high physical activity levels (PR: 0.80; 95% CI: 0.63–0.99) (Table 3).

## 4 Discussion

### 4.1 Main findings

#### 4.1.1 Prevalence of physical activity

In the present study, it was found that 54.5% of the students exhibited a high level of physical activity, while 36.6% and 8.9% reported low and moderate levels of physical activity, respectively. This finding is similar to that reported by Wafi et al., who, during the context of isolation due to the COVID-19 pandemic, found that 53.3% of medical students in Saudi Arabia maintained a high level of physical activity, while 46.7% presented moderate or low levels (41). This contrasts with the findings of a study conducted by Kosendiak et al. in Poland during the initial stage of the COVID-19 pandemic, in which 80.7% of university students were physically inactive, 10.6% were minimally active, and only 8.7% achieved high levels of physical activity (42). It also differs from the results of the study by Alrushud et al. in Saudi Arabia, conducted during the COVID-19 lockdown period, which showed that approximately 80% of medical science students were physically inactive (19). During the COVID-19 pandemic, Sidebottom et al. conducted a cross-sectional study among university students in the United States and observed a marked decrease in physical activity frequency. The median number of days per week of vigorous activity decreased from 2 to 1; moderate activity, from 4 to 1; and light activity, from 4 to 2 days per week (43). Similarly, Luethy et al., in a study conducted after the lifting of health restrictions in the U.S., reported that veterinary medicine students engaged in an average of 4 ± 5 h per week of moderate to vigorous physical activity, with a median of 3 h per week (IQR: 2–5) (44). In Latin America, Souza et al. conducted a cross-sectional study in Brazil among medical and physical education students and found that only 32.3% of medical students met physical activity recommendations (≥300 min per week), compared to 65.2% of physical education students (2). Likewise, in Chile, Merellano-Navarro et al. reported that 52.1% of university students had a high level of physical activity, 37.9% a moderate level, and 10% a low level (45). Finally, Montoya Hurtado and Cañón Buitrago, in a descriptive study conducted in Colombia and Mexico, found a general pattern of moderate physical activity: students walked an average of 0.37 h per day (approximately 22 min daily), engaged in physical activity 2.37 days per week, with an average duration of 37.3 min per session (46).

The high prevalence of low levels of physical activity during the pandemic has been associated with multiple psychosocial and structural factors. Among the main contributors are the reduction in family income, the closure of sports centers, the lack of adequate equipment, and the absence of support networks or peers that encourage regular physical exercise (41). During the pandemic, the strict lockdown measures imposed by Latin American governments severely restricted mobility and access to public and recreational spaces (47), particularly affecting students who relied on these environments to engage in physical activity (29, 48).

Additionally, the abrupt transition to remote education resulted in an increased academic workload for many students, creating additional stress related to time management and reducing opportunities for voluntary physical activity (49, 50). Prolonged screen time, along with the sedentary lifestyle associated with virtual learning, significantly contributed to the decline in physical activity levels during this period (51, 52). Finally, the conditions of the home environment during lockdown played an important role. In particular, many medical students in Latin America may have lived in small spaces with limited suitability for physical activity. The lack of basic equipment for exercising at home, along with potential overcrowding, may have contributed to decreased motivation and, consequently, lower levels of physical activity during that period (53, 54).

#### 4.1.2 Remote learning and physical activity levels

Students who reported studying remotely due to the pandemic were associated with a 19% lower prevalence of high physical activity levels, a finding consistent with similar studies conducted in various countries during the COVID-19 lockdown context. Haddad et al., in a cross-sectional study conducted in Tunisia with physical learning students, observed that the virtual modality was associated with a 10% decrease in the average number of daily steps on weekdays (*β* = −750.63; *p* = 0.050) (55). Similarly, Frömel et al., in a comparative study with high school adolescents in the Czech Republic and Poland during the COVID-19 pandemic, reported that remote learning was associated with a 94.4% reduction in the median weekly vigorous physical activity in males (from 1,080 to 60 MET-min/week) and a 100% reduction in females (from 240 to 0 MET-min/week) (56). In the Latin American context, Salazar et al., in a cross-sectional study conducted in Peru with university students during the lockdown, reported that during the implementation of remote learning, the prevalence of high-level physical activity decreased by 55.6%, moderate-level activity by 10.9%, and the proportion of students with low-level activity increased by 111.1%. Additionally, the proportion of students meeting WHO recommendations (≥150 min per week) decreased by 36.1% (57). However, these findings contrast with those reported by Almeheyawi et al. in Saudi Arabia, who identified an inverse relationship between the number of virtual class hours and physical activity levels: students attending between 25 and 30 h per week performed 277.66 MET-min/week less physical activity (95% CI: −484.65 to −70.66; *p* = 0.002), while those with more than 30 h per week performed 363.24 MET-min/week less (95% CI: −593.97 to −132.50; *p* = 0.002), compared to those studying less than 25 h (58).

This association could be explained by multiple factors at the individual, familial, and structural levels. Remote learning often involves long hours in front of screens, both for attending classes and for completing assignments or studying. This increase in screen time may have promoted sedentary behavior, reduced opportunities for physical activity, and contributed to mental fatigue, thereby decreasing motivation to exercise (12, 59, 60). In addition, the transition from an in-person to a virtual learning environment disrupts the organization of daily routines; with the elimination of commuting to campus, students lose the everyday structure that previously facilitated incidental physical activity, such as walking, climbing stairs, or participating in sports and extracurricular activities (61, 62).

Mobility restrictions and the closure of sports facilities may have limited access to spaces and equipment necessary to maintain a physically active lifestyle, particularly affecting students who relied on resources provided by universities (54, 63, 64). In Latin American households, students may live in small spaces without adequate conditions or privacy to exercise, representing a significant structural and psychological barrier to staying physically active (3, 53).

Likewise, studying from home can increase the burden of household and family responsibilities. Some students must take on caregiving duties for relatives, perform household chores, or face frequent interruptions, which reduces their available time and energy for physical activity (150). Added to this is the emotional impact of confinement, social isolation, and uncertainty about the academic and professional future, all of which raise levels of stress and anxiety. These mental health factors, strongly linked to the pandemic context, negatively affect motivation to establish and maintain a regular exercise routine (65, 66).

### 4.2 Other factors associated with physical activity level

Women were associated with an 11% lower prevalence of high physical activity levels. This finding is consistent with the results reported by Baj-Korpak et al., in a descriptive and comparative study conducted among university students in Poland and Belarus, where women showed a significantly lower prevalence of high levels of physical activity compared to men in the context of the COVID-19 pandemic (67). Similarly, Frömel et al. reported in a comparative study with high school students in the Czech Republic and Poland that there was a 94.4% reduction in the median weekly vigorous physical activity in males and a 100% reduction in females. However, in terms of total physical activity, males reduced their level by 59.1% (from 5,422 to 2,220 MET-min/week), while females had a 19.6% reduction (from 3,444 to 2,769 MET-min/week) (56). In contrast, a cross-sectional study conducted by Bertrand et al. among Canadian students found that women engaged in more moderate-to-vigorous physical activity than men both before and during the pandemic (*p* = 0.01), although activity levels decreased over time in both sexes: among women, from 89.4 ± 70 to 73.3 ± 55.3 min per week, and among men, from 72.5 ± 62.5 to 40.5 ± 35.7 min per week (68). This association could be explained by the fact that women tend to report higher levels of anxiety, worry, and hopelessness, which makes them more susceptible to psychiatric disorders such as anxiety or depression, ultimately translating into lower physical quality of life (69, 70). Additionally, the lack of privacy and adequate space at home may demotivate them from engaging in physical activity. They may also have experienced higher levels of stress and anxiety, which further reduces motivation (71). Another relevant aspect is the perception of having less free time due to multiple roles and responsibilities. Concerns about safety in the external environment, especially during the pandemic, also limited their opportunities to exercise (72).

Perceiving the pandemic as mild or not serious was associated with a 40% higher prevalence of high physical activity levels. On the other hand, in the study by Vázquez-Rodríguez, conducted among young people in the state of Tamaulipas, Mexico, no significant association was found between a poor perception of the pandemic and physical inactivity (53). Additionally, previous reports have indicated that a negative perception of the pandemic greatly influenced individuals' mood, which in turn affected adherence to physical activity routines (73). A Peruvian study conducted by Yanamango-Castillo et al. reported that, in their study population, there was no association between physical activity and the perception of the COVID-19 situation (74). This contrast in results may be due to the fact that the impact of the pandemic varied for each individual, largely depending on their personal circumstances and emotional state. These factors could be reflected in the degree of compliance with restriction measures and the motivation to engage in physical activity (75, 76). This association may be explained by the fact that students who do not perceive the pandemic as a serious threat may be less concerned about restrictions and the risks of going outside, which allows them to maintain or even increase their outdoor physical activity, such as walking, running, or playing sports (77). Furthermore, a less serious perception of the pandemic is often associated with lower levels of stress and anxiety, which facilitates greater motivation and willingness to engage in physical exercise (78). Those who consider the pandemic to be mild may also be more likely to seek out and participate in recreational and social activities that involve physical exercise, taking advantage of opportunities to stay active (79). These students tend to have a more balanced and positive approach to time management during the pandemic, integrating physical activity as a priority. Finally, it is possible that students with this perception were less affected by lockdown restrictions, more easily adapting their exercise routines to changing circumstances (3, 80). A minimized risk perception may be associated with lower adherence to lockdown measures, which could have facilitated the continuation of outdoor physical activities. However, this perception could also reflect a lower level of information or a low perception of vulnerability-factors that were not assessed in this study.

Having obesity was associated with a 31% lower prevalence of high physical activity levels. This finding aligns with the results reported by Vázquez-Rodríguez et al., in a study conducted among young adults in Poland during the COVID-19 lockdown, which found that having obesity reduced the likelihood of engaging in physical activity by 41% compared to those with normal weight (OR = 0.59) (53). Similarly, the study by Alrushud et al., conducted in Saudi Arabia during the COVID-19 pandemic lockdown, indicated that none of the medical students with obesity engaged in high levels of physical activity. In this group, 84.8% were inactive and only 15.2% reached a sufficiently active level (19). According to Alhashemi et al., in a cross-sectional study conducted in 2021 in Syria among medical students, being overweight or obese was associated with a lower prevalence of sports participation, showing a 20.4% reduction compared to students with normal weight. In fact, 79.6% of overweight students reported not engaging in sports regularly (81). Following the pandemic, this trend persisted. Herreros-Irarrázabal et al., in a multicenter cross-sectional study of medical students from eight Latin American countries, found that a higher body mass index was associated with a 13% decrease in time spent on strength training, a form of vigorous physical activity (*r* = −0.13; *p* = 0.039) (25).

This association could be explained by the consequences of home confinement caused by the pandemic, which led to weight gain due to poor dietary habits and a reduced engagement in physical activities (82, 83). From a biological perspective, medical students with obesity may face additional challenges in maintaining high levels of physical activity due to the physical limitations associated with excess body weight (84). The physical and emotional demands of their rigorous academic training may be exacerbated by the physiological consequences of obesity, such as insulin resistance, hypertension, and dyspnea, which can hinder their ability to engage in vigorous exercise (85, 86). Moreover, chronic fatigue and lack of energy, common among medical students due to long hours of studying and clinical work, can be intensified by obesity, resulting in a lower predisposition to maintain a regular and vigorous exercise routine (87, 88). The relationship between obesity and physical activity may also be bidirectional. On one hand, obesity may precede the reduction in physical activity due to associated physical and psychological limitations (89). On the other hand, a decrease in physical activity may lead to the development of obesity over time, as a result of a sedentary lifestyle and lack of regular exercise (90). Finally, from a psychosocial perspective, it is possible that students with obesity experience greater stigmatization, negative body image, or low self-efficacy, which limits their willingness to engage in physical activities, especially in group or public settings (91–93).

Diabetic students were associated with a 59% higher prevalence of high physical activity levels. This contrasts with the findings of Alrushud et al., who, during the COVID-19 pandemic in Saudi Arabia, evaluated medical students and found that among those with chronic diseases, only 6.3% reported a high level of physical activity (19). Isolation intensified the sense of fragility and vulnerability in these patients, contributing to increased inactivity (94). Similarly, Peimani et al. reported in a cross-sectional study conducted during the COVID-19 pandemic among adults with type 2 diabetes that only 29.1% achieved adequate levels of physical activity, and that diabetes-related distress (OR = 0.60) and perceived isolation (OR = 0.77) were negatively associated with engagement in physical activity (95). These results are also consistent with those reported by Andersen et al., who in a cross-sectional study conducted in the United States among adults with type 2 diabetes during the pandemic found that 31.3% of respondents reported having decreased their physical activity since the beginning of the lockdown (96). The finding in our study could be explained by the fact that physical activity is part of a healthy lifestyle and a key component in the treatment of patients with chronic diseases. During the COVID-19 pandemic specifically, it helped prevent systemic inflammation and had a positive effect on disease outcomes (97, 98). From a pathophysiological perspective, the relationship between diabetes and physical activity is based on the beneficial effects of exercise on glucose metabolism and insulin sensitivity (99, 100). Regular physical activity promotes glucose uptake by muscle tissues, thereby reducing blood glucose concentrations and improving glycemic control (101). This effect is partly due to the activation of metabolic pathways that increase the translocation of GLUT4 glucose transporters to the cell membrane, facilitating glucose entry into muscle cells (102). Moreover, acute exercise induces a series of metabolic and structural adaptations in skeletal muscle that enhance insulin sensitivity (103). These include the activation of the insulin signaling pathway and the synthesis of proteins involved in glucose transport and metabolism, as well as an increase in the number and size of mitochondria, which favors fatty acid oxidation and glucose utilization as an energy source (104). Regular physical activity is also associated with a reduction in peripheral insulin resistance, a characteristic feature of type 2 diabetes (99). This is partly due to decreased levels of circulating free fatty acids and improved endothelial function, which facilitates glucose transport from the blood into peripheral tissues (105). This finding, although relevant, should be interpreted with caution, as the number of students who reported having diabetes was low (1.2%), which may limit the generalizability of the result.

Students who reported having had COVID-19 were associated with a 21% higher prevalence of high physical activity levels. This association could be explained by various biological and psychological factors that influence individuals' behavior after the illness. From a biological perspective, those who have experienced COVID-19 may develop greater awareness of the importance of exercise for health and for strengthening the immune system (106). Given the viral nature of the disease and its impact on the respiratory system, it is plausible that individuals who have recovered from COVID-19 are motivated to engage in physical activities as part of their recovery and pulmonary rehabilitation (107, 151). Moreover, regular exercise can help improve cardiovascular and respiratory function, which may be especially relevant for those who experienced respiratory complications during the illness (108, 109). From a psychological standpoint, having overcome COVID-19 may lead to a renewed appreciation for health and well-being, as well as a desire to make the most of life after facing a potentially life-threatening experience (110). This perception of vulnerability may motivate individuals to adopt healthier lifestyle habits, including participation in physical activities, as a form of self-care and prevention of future illnesses (111). It is important to note that, to date, no previous studies have identified a direct association between having had COVID-19 and a higher prevalence of physical activity among medical students.

Trusting the government's ability to manage the COVID-19 pandemic was associated with a 21% lower prevalence of high physical activity levels. A plausible explanation is that students with higher institutional trust may have adhered more strictly to governmental restrictions—such as stay-at-home orders, mobility reductions, or avoidance of public spaces—which inadvertently limited opportunities for moderate or vigorous physical activity (97). Evidence from international studies shows that individuals with greater trust in government tend to comply more rigorously with public health policies during crises, including reduced mobility, distancing behaviors, and greater risk-avoidant conduct (112, 113). Such compliance has been consistently associated with decreased physical activity levels, particularly reductions in outdoor exercise and structured vigorous activity (114). Therefore, rather than reflecting psychological distress or lack of motivation, the lower prevalence of high physical activity observed among students who trusted governmental pandemic management may represent a behavioral response aligned with strict compliance to public health recommendations during lockdown conditions (29).

Being a student at a private university was associated with a 17% higher prevalence of high physical activity levels. In contrast, Lin Luo et al. found that in a Chinese school sample of students aged 10–20 years, the type of educational institution (private vs. public) was not significantly associated with the lack of home-based physical exercise during the COVID-19 lockdown (OR = 0.913; 95% CI: 0.729–1.144; *p* = 0.430) (115). This association may be explained by several socioeconomic, cultural, and structural factors. First, private universities, having greater financial and physical resources, may have had a higher capacity to rapidly adapt to the restrictions imposed by the pandemic (64). This includes the implementation of measures such as disinfection stations, frequent cleaning protocols, and physical distancing guidelines, which facilitated the continuation of physical activity in confined environments and allowed students to remain active safely (116). Moreover, these institutions may have developed exercise programs tailored to the new reality imposed by the pandemic, such as online or outdoor classes, providing viable alternatives for staying active while adhering to social distancing measures (117, 118). The availability of technological resources and the capacity to invest in digital infrastructure may also have facilitated the transition to virtual exercise modalities, allowing students to access workouts and classes from the safety of their homes (117, 119). In the context of the COVID-19 pandemic, it is also important to consider the socioeconomic background of students attending private universities (120, 121). These students tend to come from families with higher educational and socioeconomic levels, which may influence their ability to maintain healthy lifestyle habits, including physical activity, during the health crisis (29). Families with greater economic resources may have more options to adapt to pandemic-related restrictions, such as the ability to invest in home exercise equipment, subscriptions to online classes, or access to safe outdoor spaces for physical activity (122). Additionally, economic stability can reduce financial stress and provide a more favorable environment for prioritizing health and well-being, including regular participation in physical activity (123, 124).

Being at moderate/high risk of tobacco use was associated with a 20% lower prevalence of high physical activity levels. This contrasts with findings reported by Romero-Blanco et al. in a longitudinal study conducted during the COVID-19 lockdown among Spanish university students, where it was observed that smokers significantly increased their weekly physical activity, from 253.6 to 524.7 min per week (difference: +271.1 min; 95% CI: 46.5–495.8; *p* = 0.020) (80). Similarly, prior to the pandemic, Barroso et al., in a cross-sectional study conducted among U.S. high school students between 2015 and 2021, observed that each additional day with at least 60 min of physical activity was associated with a 7% reduction in the likelihood of smoking cigarettes (aOR = 0.93; 95% CI: 0.90–0.95), highlighting the importance of physical activity as a protective factor against tobacco use in adolescents (125). Additionally, according to Yu et al., in a cross-sectional study conducted in 2018 that included Chinese residents aged 16 and older, physical activity was associated with a 28% reduction in the odds of smoking (OR = 0.718; 95% CI: 0.673–0.765) prior to the pandemic (126). This finding could be explained by considering that moderate/high risk of tobacco use—assessed via a self-reported index—is commonly associated with unhealthy lifestyles and dysfunctional coping behaviors. Several studies have documented that smoking often coexists with other harmful practices, such as physical inactivity, poor diet, and substance use, particularly in contexts of chronic stress such as that generated by the pandemic (127, 128). In the case of medical students, this period was marked by high levels of anxiety, academic burden, and disruptions in emotional well-being (129). Smoking, even at a risk stage, can exacerbate these issues by impairing pulmonary and cardiovascular function and contributing to symptoms such as dyspnea, chronic fatigue, and decreased energy levels (130, 131). These physiological effects, combined with emotional factors such as stress and frustration, may have reduced the motivation to maintain high levels of physical activity (132, 133).

### 4.3 Relevance of findings in public health

These findings are relevant to public health as they highlight the adverse impact that virtual learning can have on physical activity among university students—a key group for the primary prevention of non-communicable diseases. They also reveal gender inequalities and clinical factors (such as obesity and diabetes) that may influence physical activity levels, as well as the influence of social and political perceptions on healthy behaviors (134, 135). These results underscore the need to design, implement, and evaluate integrated and culturally relevant interventions that promote physical activity even in remote learning contexts, taking into account the interaction of individual, social, clinical, and political determinants (136). Furthermore, they emphasize the importance of contextualizing these findings within public health disaster scenarios, such as epidemics, pandemics, and outbreaks (137). Such strategies could include home-based online physical activity programs, gender-sensitive awareness campaigns, university policies that encourage exercise during the virtual academic day, and the strengthening of self-care capacities among students, particularly during public health emergencies (66, 138, 139). Incorporating these interventions into public health planning and university management is essential to protect and improve the health and well-being of university students, who represent the next generation of the country's workforce and human capital.

### 4.4 Limitations and strengths

This study presents several limitations. First, its cross-sectional design prevents the establishment of causal relationships between the variables evaluated. Second, the non-probabilistic snowball sampling strategy may have led to selection bias, potentially over-representing students who are more active in academic or scientific networks, which could limit the generalizability of the findings. Third, the main outcome—physical activity level—was measured using the self-reported IPAQ-S, which is subject to recall bias and social desirability bias; participants may have over- or under-reported their actual activity levels to align with socially acceptable behaviors. Additionally, although the academic year was collected, it could not be used to reliably classify students into preclinical or clinical stages across countries due to heterogeneity in medical curricula in Latin America; therefore, this variable was not included as a covariate in the multivariable model. This limitation may have prevented exploration of potential differences in physical activity by study stage. Furthermore, the possibility of unmeasured confounding must be considered, as potentially relevant variables associated with physical activity, previously reported in the literature, were not included. For example, sleep quality has been positively associated with the frequency and duration of physical activity (140–142); diet quality or nutritional status, since a healthy diet promotes physical and mental readiness to engage in regular physical activity (143, 144); and social support, which is a key determinant for participation in physical activities, especially among university students (145–147). Moreover, prior participation in organized sports has been associated with lower physical activity levels during the pandemic, as those who relied on group training and specific facilities were unable to replace them at home (148, 149). The absence of measurement of these variables may have introduced residual confounding, affecting the estimated associations between sociodemographic and clinical factors and physical activity levels in this university population during the pandemic.

One of the main strengths of this study is that it is among the first to evaluate the association between remote learning and physical activity levels in medical students from Latin America during the first wave of the COVID-19 pandemic. This approach provides valuable regional evidence for understanding the effects of virtual learning on the healthy lifestyles of future health professionals-key information for designing learning interventions and policies tailored to public health emergency contexts. The study included a considerable sample size of students from various Latin American countries and employed rigorous statistical methods to analyze associations. Finally, additional relevant factors such as sex, perception of the pandemic, obesity, diabetes, tobacco use, and type of university were explored, allowing for a more comprehensive view of the determinants of physical activity in this group during the global health crisis.

## 5 Conclusions

These findings underscore the need for university-based interventions specifically designed to mitigate the negative impact of remote or hybrid learning on physical activity levels. Strategies should address structural and behavioral barriers, integrate physical activity promotion into virtual curricula, and ensure accessibility of exercise opportunities for students with health vulnerabilities or risky behaviors. In practical terms, universities and health professionals may support these goals by incorporating brief exercise modules into virtual classrooms, offering telehealth-based counseling on lifestyle habits, and developing gender-sensitive or socially tailored interventions. Digital tools—such as mobile apps, wearable devices, or online exercise programs—may also serve as low-cost, scalable resources to promote active behaviors during periods of restricted mobility.

Future research should explore these relationships through longitudinal designs to better assess causality and temporal changes in physical activity across different stages of the medical curriculum. Comparative studies across Latin American countries are also needed to elucidate contextual determinants and evaluate the effectiveness of university-based interventions. Additionally, examining the role of environmental, socioeconomic, and psychosocial factors—including sleep, nutrition, and social support—may provide a more comprehensive understanding of the mechanisms influencing physical activity in this population.

## Data Availability

The raw data supporting the conclusions of this article will be made available by the authors, without undue reservation.
