# Correction: Remote learning and physical activity in medical students from Latin America: a cross-sectional study during the first wave of the COVID-19 pandemic

**DOI:** 10.3389/fspor.2026.1791632

**Published:** 2026-02-02

**Authors:** Mario J. Valladares-Garrido, Renzo Acosta-Porzoliz, Alejandro Juarez-Ubillus, Milagros Diaz-Torres, Ludwing A. Zeta Solis, David Astudillo Rueda, Fatima Jiménez-Mozo, C. Ichiro Peralta Chiguala, Christopher G. Valdiviezo-Morales, E. Sebastian Benavides Alburqueque, Estrella Christabel Porras Núñez, Víctor J. Vera-Ponce, César J. Pereira-Victorio, Carlos Culquichicón

**Affiliations:** 1 Escuela de Medicina Humana, Universidad Señor de Sipán, Chiclayo, Peru; 2 Facultad de Medicina Humana, Universidad Peruana Cayetano Heredia, Lima, Peru; 3 EpiHealth Research Center for Epidemiology and Public Health, Lima, Peru; 4 Facultad de Medicina Humana, Universidad de San Martín de Porres, Chiclayo, Peru; 5 Facultad de Medicina, Universidad Nacional de Piura, Piura, Peru; 6 Escuela de Medicina, Universidad Cesar Vallejo, Piura, Peru; 7 Universidad Nacional Federico Villarreal, Lima, Perú; 8 Facultad de Medicina (FAMED), Universidad Nacional Toribio Rodríguez de Mendoza de Amazonas (UNTRM), Amazonas, Peru; 9 Facultad de Medicina, Universidad Continental, Lima, Perú; 10 University of Washington, School of Public Health, Seattle, WA, United States

**Keywords:** COVID-19, cross-sectional study, Latin America, medical students, physical activity, remote learning

Christopher G. Valdiviezo-Morales, E. Sebastian Benavides Alburqueque, and Estrella Christabel Porras Núñez were erroneously assigned to affiliation 6, Escuela de Medicina, Universidad Cesar Vallejo, Piura, Peru. The correct affiliation is 5, Facultad de Medicina, Universidad Nacional de Piura, Piura, Peru.

The original version of this article has been updated.

